# Bibliometric analysis of potassium channel research

**DOI:** 10.1080/19336950.2019.1705055

**Published:** 2019-12-19

**Authors:** Jingjing Shi, Shuqing Shi, Shuai Shi, Qiulei Jia, Guozhen Yuan, Yuguang Chu, Huan Wang, Yuanhui Hu, Hanming Cui

**Affiliations:** a Guanganmen Hospital, China Academy of Chinese Medical Sciences, Beijing, China; b Graduate School of Beijing University of Chinese Medicine, Beijing, China

**Keywords:** Bibliometric, potassium channel, ﻿Cite﻿Space, visual analysis

## Abstract

ABSTRACT Objective: To explore the research status, hotspots, and trends in research on potassium channel. Methods: The Web of Science core collection database was used as the data source and the visual analysis software Citespace5.4 R3 was used to visualize the studies of potassium channel in the past 10 years. The national/institutional distribution, journal distribution, authors, and related research were discussed. Results 17,392 articles were obtained. The USA, Peoples R China, Germany, England, and Japan were the main countries in the field and University of California was the most important institution for the study of potassium channel. PLoS One was the most productive journal and proceedings of the national academy of sciences of the united states of america was the most frequently cited journal in potassium channel research. The author with the highest number was Colin G Nichols and the author with the highest co- cited frequency was Sanguinetti MC. The three hot spots of potassium channel research were gene expression, Ca2+ activated k+ channel and nitric oxide. The top four research frontiers of potassium channel research were bk channel,blood pressure,oxidative stress and electrophysiology. Conclusion The study provides a perspective for understanding the potassium channel research and provides valuable information for potassium channel researchers to identify potential collaborators, partner institutions, hot topics and research frontiers.

Potassium channels are a diverse and ubiquitous family of membrane proteins that allow rapid and selective flow of K+ ions across the cell membrane, and thus generate electrical signals in cells. Potassium channels are found in most cell types and control a wide variety of cell functions [1]. K+ channels include four of the 11 families of the voltage-gated ion channel superfamily: ①Voltage-gated K+ channels (Kv); ② Ca2+ – and Na+ – activated K+ channels (KCa, KNa); ③inwardly rectifying K+ channels (Kir); and ④ two-pore domain K+ channels (K2P) [2].

Potassium channels commonly play a major part in the repolarization of action potentials [3], and it can regulate the secretion of hormones and neurotransmitters and establish cell plasma membrane potential. This large family can be regulated by voltage, Ca2+, neurotransmitters and the signaling pathways that they stimulate.

Potassium (K+) channels participate in many physiological processes, cardiac function, cell proliferation, neuronal signaling, muscle contractility, immune function, hormone secretion, osmotic pressure, changes in gene expression, and are involved in critical biological functions, and in a variety of diseases [4].

Bibliometric analysis has been widely used in various areas to estimate the productivity of institutions, countriesinstitutions,countries, and authors; identify international collaborations and geographic distributions; and explore research hotspots and frontiers in specific fields [5,6]. CiteSpace is one of the bibliometric visualization tools for visualizing and analyzing emerging trends and transition patterns in scientific literature, which was developed by Chaomei Chen in 2004 [7,8]. Numerous journals have published articles on the potassium channel.However, to date, no bibliometric studies regarding the trends in potassium channel research activity over the past few decades have been published and there has been a consequent lack of attention to this field. The purpose of our study was to provide researchers with some direction regarding potassium channel research using bibliometrics.

The data for analysis were extracted from the Science Citation Index Expanded (SCI-expanded) of Web of Science Core Collection (WoSCC) bibliographic database. To ascertain the trend in publications, we collected 10 years’ worth of data from 2009 to 2018. The data were downloaded directly from the database as secondary data without further animal experiments; therefore, no ethical approval was required.

The data search was conducted on 31 July 2019 and collected in 1 day to avoid any potential bias due to the daily updating of the database. The search keywords entered into the database were as follows: TS = (potassium channel* OR potassium ion channel* OR KATP channel*) and language: (English) and document types: (article). Seventeen thousand three hundred and ninety-two articles were obtained, and 228 proceedings papers (1.311%), 51 book chapters (0.293%), 6 retracted publications (0.034%) and 1 data paper (0.006%) were contained.

The downloaded data were analyzed based on the Web of Science database literature analysis report and export information function. Then we used Microsoft 2018 to count the number of publications every year. Citespace5.4 R3 was used to analyze and construct knowledge maps. Visualization knowledge maps consist of nodes and links. Different nodes in a map represent elements such as an institution, country, author, and keyword, links between nodes represent relationships of collaboration/co-occurrence or cocitations. The color of nodes and lines represents different years. The purple round represents that nodes with high centrality which are usually regarded as turning points or pivotal points in a field [9,10].

## General information and annual publication output

**Figure 1.**
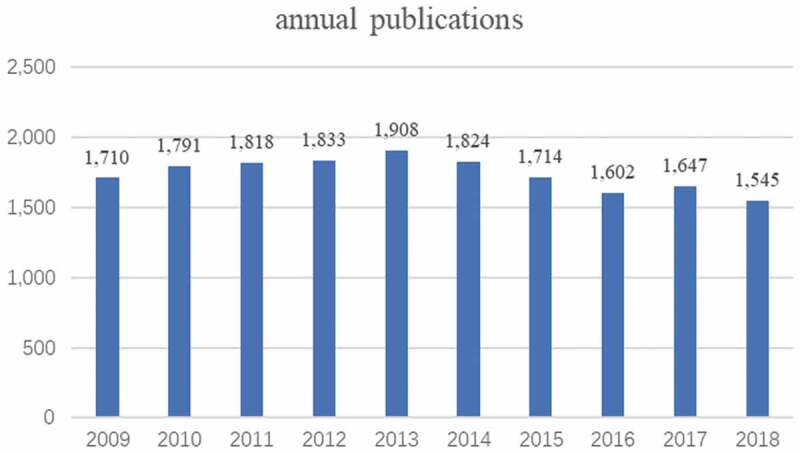
The number of annual publications on potassium channel research from 2009 to 2018.

Seventeen thousand three hundred and ninety-two articles were obtained, to explore the trends in potassium channel research, we visualized the yearly outputs of relevant articles . As shown in Figure 1 the trend of world potassium channel research publications remained stable high in the past 10 years, the average annual publications are 1,739.

## Active countries and institutions

**Figure 2.**
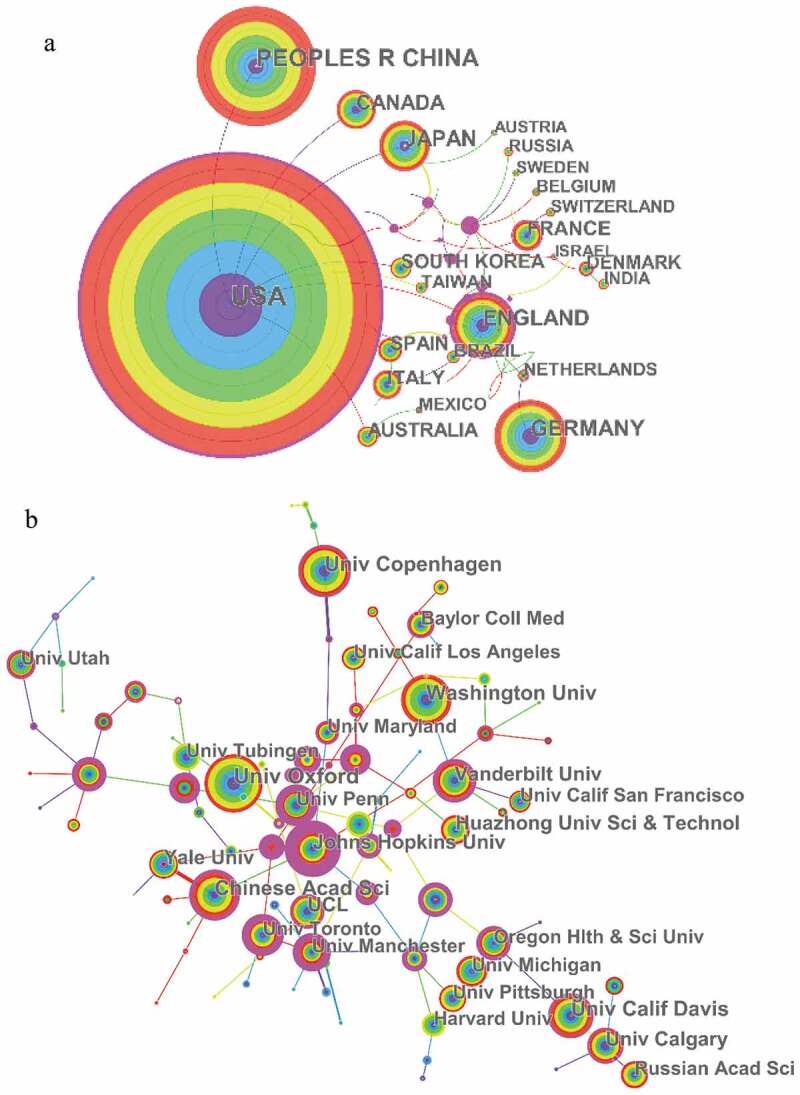
The analysis of countries and institutions. (a) Network of countries/territories engaged in potassium channel research; (b) Network of institutions engaged in potassium channel research.

Distribution maps provide valuable information and help researchers to identify potential collaborators [11].The data showing the publication contributions of different countries and institutions are shown in Table 1, while the connection between countries or institutions is shown in the network (Figure 2(a,b)).

**Table 1.** The top 10 countries and institutions contributed to publications on potassium channel research.

Rank	Country/Territory	Frequency N = 17,392	Institution	Frequency N = 17,392
1	USA	6,616	University of California	771
2	Peoples R China	2,722	Center national de la recherche scientifique	405
3	Germany	1,700	Institut National de la Sante et de la Recherche Medicale (INSERM)	360
4	England	1,415	University of London	336
5	Japan	1,223	University of Texas System	327
6	Canada	951	University of Oxford	291
7	France	763	Chinese Academy of Sciences	250
8	Italy	758	Pennsylvania Common wealth System of Higher Education Pcshe	247
9	Spain	631	University of Copenhagen	247
10	South Korea	575	Washington University	237

Countries and institutions engaged in potassium channel research were distributed worldwide. The 17,392 articles on potassium channel research were published by more than 7000 research institutions in 106 countries/territories (Figure 2a). The USA, Peoples R China, Germany, England, and Japan were at the top of the list. USA (6,616 articles) and Peoples R China (2,722articles) were the top two countries. Figure2(a) shows that the United States attached great importance to cooperation, and had close cooperation with China, Canada, Japan, South Africa, Britain, and Australia. Figure 2(b) shows that most of the publications were published by American institutions (Figure 2 (b)), with University of California produced the highest number of publications on potassium channels (177), followed by Center national de la recherche scientifique (405) and Institut National de la Sante et de la Recherche Medicale (INSERM) (360) .

## Active journals and co-cited journal

Seventeen thousand three hundred and ninety-two articles have been published in 2,265 journals, there are 10 journals with publication volume greater than 100.The top 10 journals in terms of the number of publications are indicated in Table 2. The journal Plos One had the highest number at 721 (4.15%) (IF = 2.776,2018), The Journal of biological chemistry published 451 papers (2.59%) (IF = 4.106, 2018) in potassium channel research. The Journal of neuroscience ranked third at 370 papers (2.13%) (IF = 6.074, 2018).

**Table 2.** The Top 10 journals that published articles on potassium channel research.

Rank	Journal	Frequency (%) N = 17,392	IF 2018	Country Affiliation
1	PLoS One	721(4.15%)	2.776	United State
2	The Journal of biological chemistry	451(2.59%)	4.106	United State
3	Journal of neuroscience	370(2.13%)	6.074	United State
4	Proceedings of the National Academy of Sciences of the United States of America	340(1.96%)	9.580	United State
5	Journal of Physiology-London	311(1.79%)	4.950	England
6	Scientific reports	264(1.52%)	4.011	England
7	European journal of pharmacology	259(1.49%)	3.170	Netherlands
8	Journal of general physiology	234(1.35%)	4.258	United States
9	Journal of neurophysiology	217(1.25%)	2.614	United States
10	Biophysical journal	198(1.14%)	3.665	United States

**Figure 3.**
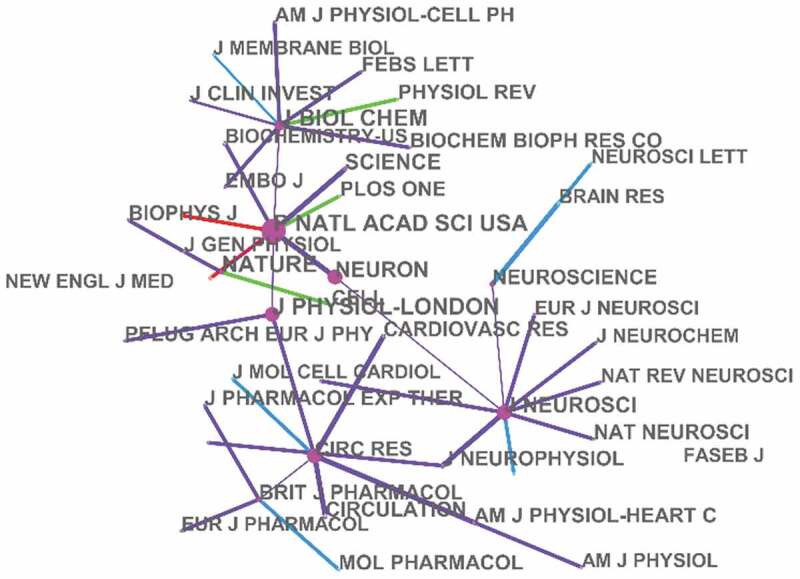
Network of co-cited authors engaged in potassium channel research.

As for the co-cited journals (Figure 3), proceedings of the national academy of sciences of the united states of america (10,446) was the most frequently cited journal in potassium channel research, the second was The Journal of biological chemistry (9408), followed by Nature (9316).

## Active authors, co-cited authors

Knowledge maps can provide information on influential research groups and potential collaborators and can help researchers to establish collaborations [12].

**Figure 4.**
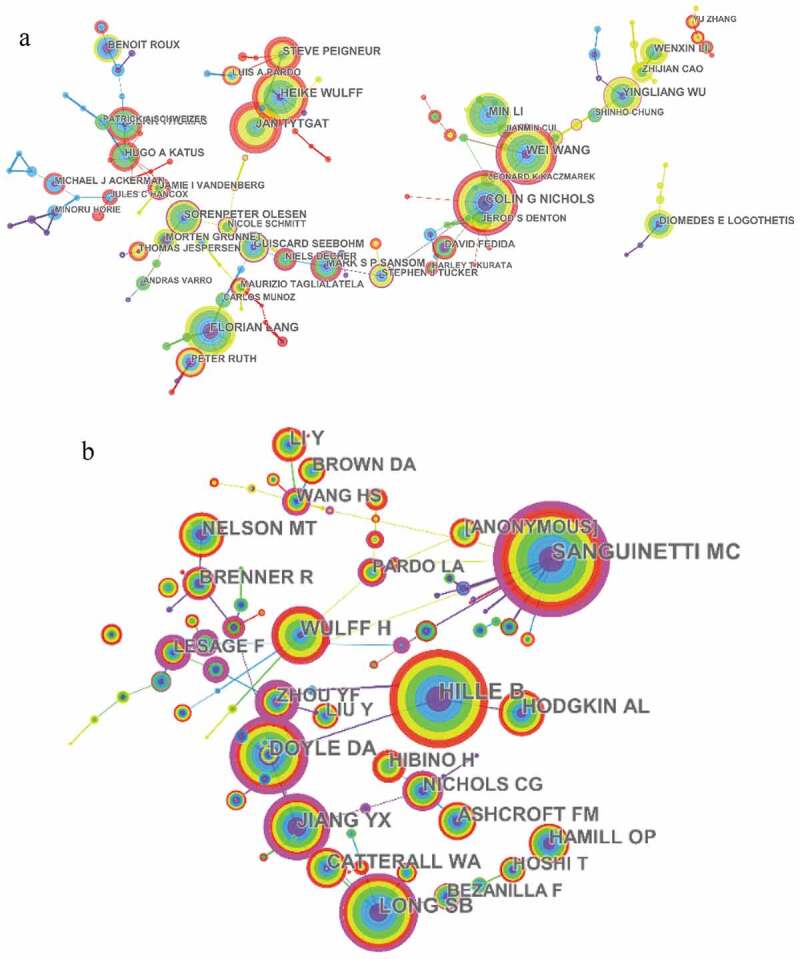
The analysis of authors. (a) Network of authors contributed to potassium channel research; (b) Network of co-cited authors engaged in potassium channel research.

Approximately 57,811authors contributed 17,392 articles related to potassium channel research. The networks shown in Figure 4(a) indicate the cooperation among authors. Colin G Nichols was the most prolific in terms of publications on potassium channel research (66 papers), followed by Wei Wang (62 papers) and Heike Wulff (59 papers). There was also a wide distribution of co-cited authors in the field of potassium channels. The connection network between co-cited authors was measured using CiteSpace V (Figure 4(b)).The papers published by Sanguinetti MC had the highest number of co-citations (1134 papers), followed by Hille B (1092 papers) and J. Long SB (812papers) (see Table 3).

**Table 3.** The top10 active authors,co-cited authors (CA) in potassium channel research.

Rank	Author	Freq	CA	Freq
1	Colin G Nichols	66	Sanguinetti MC	1134
2	Wei Wang	62	Hille B	1092
3	Heike Wulff	59	Long SB	812
4	Angela Vincent	55	Doyle DA	744
5	Florian Lang	55	Wulff H	651
6	Jan Tytgat	52	Jiang YX	625
7	Min Li	47	Hodgkin AL	552
8	Yingliang Wu	41	Nelson MT	519
9	Sergey Shabala	40	Hamill OP	487
10	Steve Peigneur	39	Catterall WA	480

## Research area analysis

**Figure 5.**
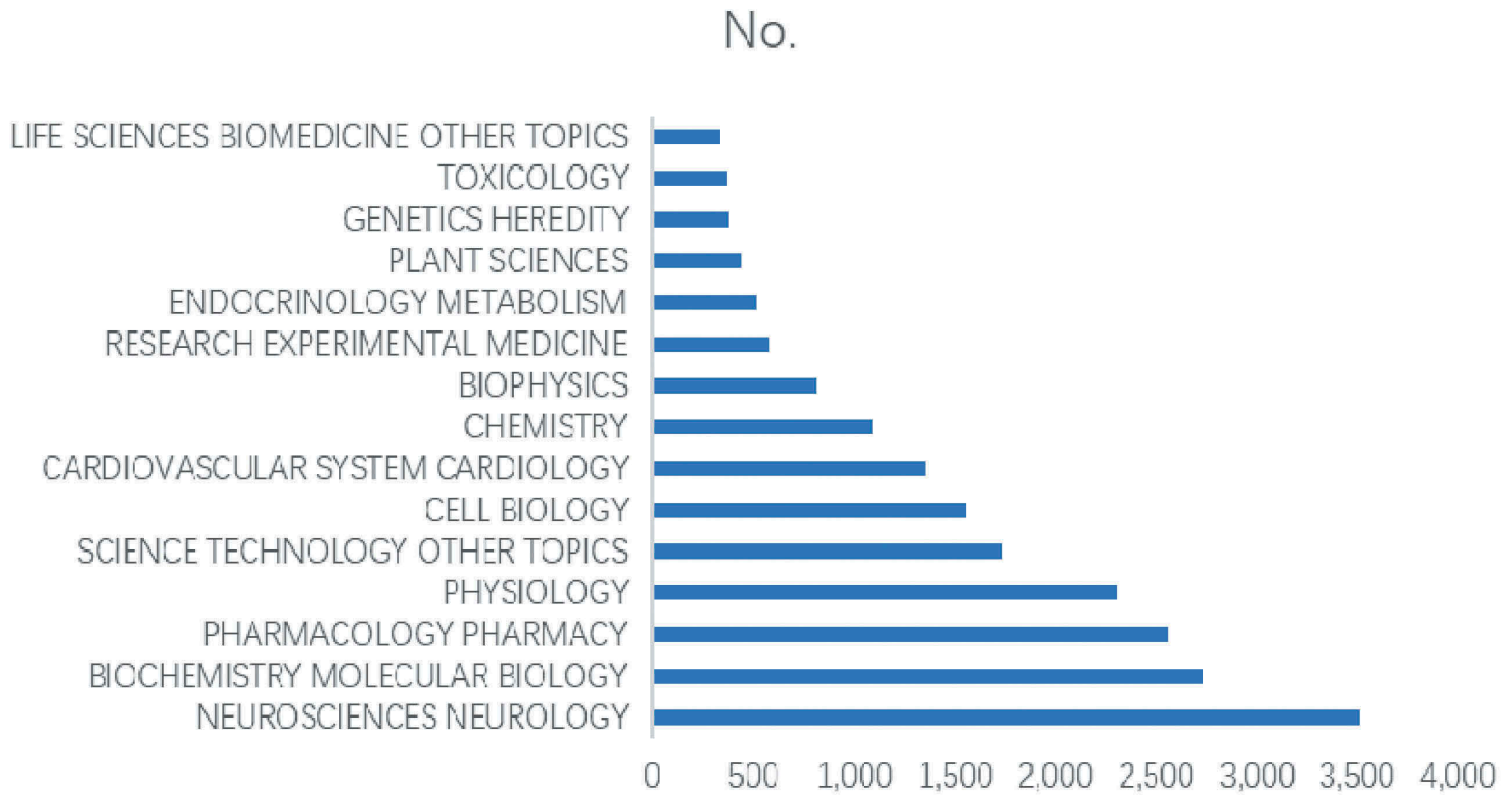
The 15 research areas on potassium channel research.

Figure 5 shows the 15 research areas that most frequently appeared in publications related to potassium channel research from 2009 to 2018. Neurosciences accounted for the largest number of publications, followed by biochemistry and molecular biology and physiology.

## Keyword co-occurrence and burst

**Figure 6.**
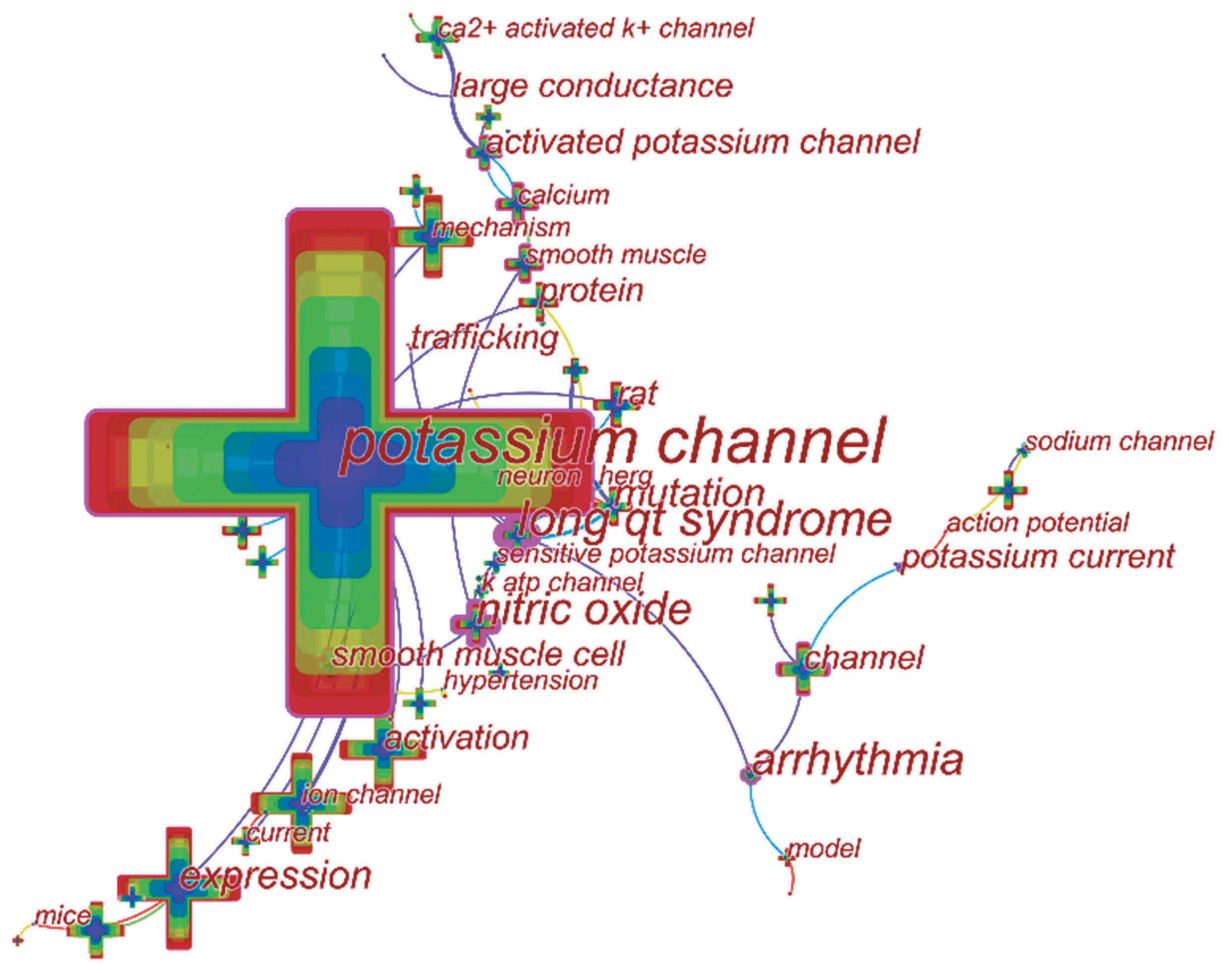
The analysis of keywords in potassium channel research.

Keywords provide a reasonable description of research hotspots, whereas burst words represent new research frontiers [7]. CiteSpace V was used to construct a knowledge map of keyword co-occurrence (Figure 6) and identified the top 20 keywords in potassium channel research articles from 2009 to 2018 (Table 4), according to frequency. The top keywords were “potassium channel,” “ion channel” “expression,” “mechanism” “cell” “protein” “rat,” “ca2+ activated k+ channel” and “nitric oxide” . Therefore, research hotspots can be summarized into the following aspects: Gene expression, ‘ca2+ activated k+ channel,’ and “nitric oxide”.

**Table 4.** Top 20 keywords in terms of frequency in potassium channel research.

Rank	Keyword	Frequency		Rank	Keyword	Frequency
1	potassium channel		7546	11	inhibition	752
2	ion channel		2499	12	calcium	699
3	expression		1819	13	mutation	699
4	activation		1407	14	modulation	664
5	mechanism		1279	15	activated potassium channel	658
6	cell		991	16	long qt syndrome	624
7	rat		831	17	in vitro	615
8	protein		809	18	crystal structure	615
9	ca2+ activated k+ channel		808	19	receptor	603
10	nitric oxide		774	20	smooth muscle	590

**Figure 7.**
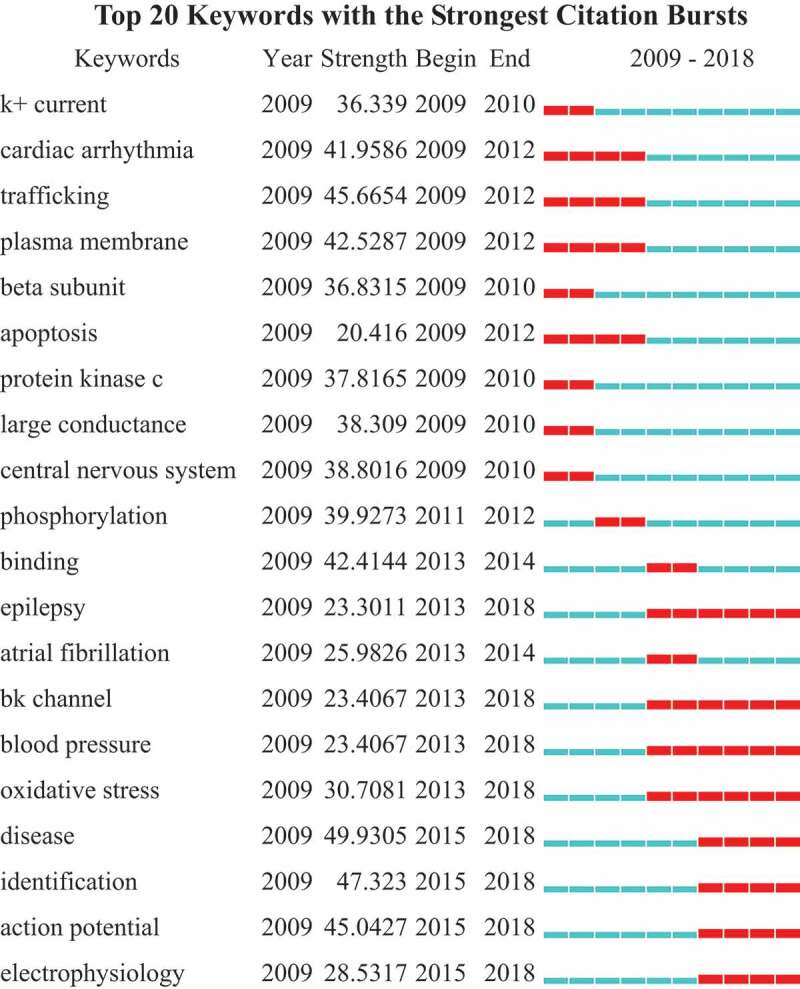
The keywords with the strongest citation bursts of publications in potassium channel research.

Keywords were identified and analyzed using strong citation bursts (Figure 7) to explore the frontiers of research. We depicted the time intervals a blue line and the time period that represents a burst keyword category as a red line, indicating the beginning and the end of the time interval of each burst [13]. As shown in Figure 7, the keywords that had strong bursts after 2014 were “bk channel” “blood pressure” “oxidative stress” “disease”, “identification”, “action potential” and “electrophysiology”. The top four research frontiers of potassium channel research were as follows: 1. bk channel 2. blood pressure 3. oxidative stress 4. electrophysiology.

According to bibliometrics analysis, the trend of world potassium channel research publications remained stable high in the past 10 years. The USA, Peoples R China, and Germany were the top countries that contributed to publications on potassium channel research. Cooperation between countries or institutions can promote the development and progress of research. The USA had close cooperation with China, Canada, Japan, South Africa, Britain, and Australia and made significant contributions to potassium channel research. University of California produced the highest number of publications on potassium channels.

The impact factor (IF) of a journal is an important factor in evaluating its value and that of included articles [14].In the top 10 published journals, “Proceedings of the National Academy of Sciences of the United States of America” had the highest impact factor (9.580), followed by the Journal of neuroscience (6.074) and Journal of physi-london (4.950). Therefore, it is a challenge to publish more high impact factor papers on potassium channel research. Through the analysis of the research areas, we found that potassium channels are most studied in Neurosciences, biochemistry and molecular biology and physiology.

Of the top 10 authors identified in this analysis, each contributed to more than 39 papers. In the network of authors contributed to potassium channel research, the largest node was (Colin G Nichols 66 articles), indicating that his important role in potassium channel research. Colin G Nichols mainly focused on the structural changes of potassium ion gated channels [15], the changes of potassium ion channels in cardiovascular diseases [16,17], nervous system diseases [18] and endocrine system diseases [19]. Wang Wei was also highly published. He and his colleagues did not cooperate much. Their research was mainly published in 2014 and 2016. His research focused on the effect of the potassium channel on neuropathic pain [20] and the contribution of TWIK-1 channels to astrocyte K+ current [21,22]. The number of citations of the top 10 co-cited authors was at least 480, and the top one was Sanguinetti MC whose studies implicated dysfunction of Ikr channels in long-QT syndrome [23]. The number of publications and co-citations of Wulff H was both high, indicating that he attaches importance to both quantity and quality. The main research direction of Wulff H was molecular properties and physiological roles of ion channels in the immune system [24]. His review discusses pharmacological strategies for targeting K(V) channels with venom peptides, antibodies, and small molecules, and highlights recent progress in the preclinical and clinical development of drugs targeting the K(V)1 subfamily, the K(V)7 subfamily (also known as KCNQ), K(V) 10.1 (also known as EAG1 and KCNH1) and K(V) 11.1 (also known as HERG and KCNH2) channels [25]. Nichols C G, Wang Wei, and Wulff H might be good candidates for research collaboration in this field.

Through the keyword cluster analysis, research hotspots can be summarized into the following aspects:

1.Gene expression, the differential expression of the potassium channel gene is related to the occurrence of various diseases. Researches show that mutations in KCNJ10 cause a specific disorder, consisting of epilepsy, ataxia, sensorineural deafness, and tubulopathy, and possibly also play a major role in blood-pressure maintenance and its regulation [26].

2.Ca2+ activated k+ channel, Calcium-activated potassium channels are potassium channels gated by calcium [27], or that are structurally or phylogenetically related to calcium-gated channels. According to the sequence homology of transmembrane hydrophobic cores,Calcium-activated potassium channels are divided into three subtypes: BK channel, IK channel and SK channel [28].

3.Nitric oxide, KATP activation associated with increased nitric oxide concentrations and inducible nitric oxide synthase induction is a key factor in cardiovascular and cerebrovascular diseases [29]. Studies have shown that Salvinorin A dilates cerebral arteries via activation of nitric oxide synthase, adenosine triphosphate-sensitive potassium channel, and the κ opioid receptor [30].

The frontiers of potassium channel research were predicted using the strongest citation bursts of publications. The three research frontiers of potassium channel research were as follows:

1. BK channel, the high-conductance calcium-activated potassium channel (BK channel) is a very complex ligand-gated potassium channel [31,32]. It is also regulated by voltage and intracellular calcium ion concentration, linking the cellular calcium signal system and membrane potential to form a negative feedback regulation, which plays a key role in many important physiological processes including smooth muscle contraction and neurotransmitter release [33].
2. Blood pressure, small-conductance (KCa2.1–2.3) and intermediate-conductance (KCa3.1) calcium-activated K(+) channels are critically involved in modulating calcium-signaling cascades and membrane potential in both excitable and nonexcitable cells [34]. Potassium homeostasis plays an essential role in the control of blood pressure [35]. One study found that the altered ATP-sensitive potassium channels may be related to the obesity-triggered increase in blood pressure [36].
3. Oxidative stress is suspected to be important in cardiovascular diseases, neurodegenerative diseases, cancerdiseases, cancer, and other aging-associated diseases. Mitochondrial ATP-sensitive potassium channels [mito(KATP)] play a critical role in modulating intracellular ROS [37]. The study revealed that renin-angiotensin system overactivation is involved in the aging process in several tissues by increasing oxidative damage and inflammation, activation of mitochondrial ATP-sensitive potassium channels [mitoK(ATP)] may play a major role in the angiotensin II-induced effects on aging and neurodegeneration [38].
4. Electrophysiology, potassium channels play an important role in regulating membrane potential and excitability of cells. With the development of electrophysiological technology, the molecular structure and functional characteristics of potassium channels have been revealed gradually.

Data on potassium channel publications were collected and retrieved from the Web of Science Core Collection database, and the analysis was relatively sophisticated and objective. A limitation of our bibliometric analysis was that, compared with papers published several years ago, recent articles did not have a high citation count. Nevertheless, CiteSpace is a useful tool for further research into potassium channels.

## Conclusions

Our study has demonstrated that numerous countries' institutions and authors have focused on potassium channel research and a lot of literature has been published. Bibliometric analysis of the literature on the potassium channels was important in allowing researchers to identify cooperations, find research hotspots and predict the frontiers of potassium channel research.
